# Cilia-related protein SPEF2 regulates osteoblast differentiation

**DOI:** 10.1038/s41598-018-19204-5

**Published:** 2018-01-16

**Authors:** Mari S. Lehti, Henna Henriksson, Petri Rummukainen, Fan Wang, Liina Uusitalo-Kylmälä, Riku Kiviranta, Terhi J. Heino, Noora Kotaja, Anu Sironen

**Affiliations:** 1 Natural Resources Institute Finland (Luke), Green Technology, FI-31600 Jokioinen, Finland; 2 Institute of Biomedicine, University of Turku, FI-20520 Turku, Finland; 3 Department of Endocrinology, Division of Medicine, University of Turku and Turku University Hospital, FI-20520 Turku, Finland

## Abstract

Sperm flagellar protein 2 (SPEF2) is essential for motile cilia, and lack of SPEF2 function causes male infertility and primary ciliary dyskinesia. Cilia are pointing out from the cell surface and are involved in signal transduction from extracellular matrix, fluid flow and motility. It has been shown that cilia and cilia-related genes play essential role in commitment and differentiation of chondrocytes and osteoblasts during bone formation. Here we show that SPEF2 is expressed in bone and cartilage. The analysis of a *Spef2* knockout (KO) mouse model revealed hydrocephalus, growth retardation and death prior to five weeks of age. To further elucidate the causes of growth retardation we analyzed the bone structure and possible effects of SPEF2 depletion on bone formation. In *Spef2* KO mice, long bones (tibia and femur) were shorter compared to wild type, and X-ray analysis revealed reduced bone mineral content. Furthermore, we showed that the *in vitro* differentiation of osteoblasts isolated from *Spef2* KO animals was compromised. In conclusion, this study reveals a novel function for SPEF2 in bone formation through regulation of osteoblast differentiation and bone growth.

## Introduction

Skeletogenesis occurs through endochondral and intramembranous ossification. During intramembranous ossification, mesenchymal stem cells (MSC) directly differentiate into osteoblasts. In endochondral ossification, MSCs first differentiate to chondrocytes forming the cartilage, which is subsequently replaced by bone. Invading vasculature brings bone forming osteoblasts to endochondral bone, where they replace the cartilage by bone^1–3^. Osteoclasts are bone resorbing cells and together with osteoblasts they are responsible of bone remodeling^4^. Bone formation is a highly controlled process and several transcription factors are required for osteoblast and chondrocyte differentiation. Runt-related transcription factor 2 (*Runx2*), Osterix (*Osx*) and Activating transcription factor 4 (*Atf4*) regulates osteoblast differentiation^5,6^, while transcription factors *Runx2*, SRY-Box-9 (*Sox9*), *Sox5* and *Sox6* are involved in the regulation of chondrocyte differentiation^7^. Disruption of *Osx* has been shown to cause complete absence of osteoblasts and mineralized matrix in mice^8^. *Runx2* is expressed specifically in MSCs which are committed to osteoblast or chondrocyte lineages, and later during development *Runx2* is expressed in osteoblasts^9^ where it is required for the expression of the osteocalcin (*Ocn*) at the onset of mineralization^10–12^. Alkaline phosphatase (*Alp*) is also required for mineralization and is expressed in osteoblasts^10,13^. In addition, *Runx2* regulates the differentiation of pre- and hypertrophic chondrocytes^7^ and *Ocn* is also expressed from the prehypertrophic chondrocytes of the growth plate^14^. Bone formation has also been shown to be dependent on cilia related signaling pathways^15–18^. Primary cilia are non-motile microtubule based structures protruding from the cell surface. This structure senses and transduces extracellular signals, which are vital for normal development of multiple organs. Bone forming osteoblasts, chondrocytes and bone matrix embedded osteocytes has been shown to possess primary cilia^19–21^. The microtubule-dependent transport mechanism called intraflagellar transport (IFT) and relative pathways are essential for correct signal transduction during skeletogenesis^15,17,18,22^. It has been shown that commitment and differentiation to chondrocytes and osteoblasts are dependent on cilia and cilia-related genes^23^ and e.g. blocking of the primary cilia formation using IFT88- siRNA in the MSCs caused the loss of cell adhesion and cell type specific differentiation^23^. Depletion of the IFT complex proteins, IFT20 and IFT80 in osteoblasts caused decreased bone mass and impaired osteoblast differentiation^24,25^, indicating an important role for functional IFT in osteoblast differentiation. Both osteoblasts and cartilage forming chondrocytes are derived from MSC, which have also been shown to express primary cilia on their surface^23,26^. Sperm flagellar protein 2 (SPEF2) is expressed in various ciliated tissues and its relevance for functional cilia has been established. *Spef2* has multiple isoforms; 5 isoforms have been identified in mice (ENSMUSG00000072663) and 14 isoforms in human (ENSG00000152582) according to Ensembl database (www.ensembl.org). Mutation in the testis specific isoform of *Spef2* by L1 retrotransposon insertion into intron 30 causes the immotile short-tail sperm (ISTS) defect in pigs, which is characterized by short and disorganized sperm tail structures causing male infertility^27,28^. In big giant head (*bgh*) mouse model, two mutations in *Spef2* gene have been characterized; missense mutation in exon 3 and nonsense mutation in exon 28. *Bgh* mice have similar spermatogenetic phenotype as the ISTS pigs, which is most likely caused by the nonsense mutation in exon 28. In addition, the *bgh* mice show primary ciliary dyskinesia (PCD)-like symptoms including sinusitis and hydrocephalus^29^, which are most likely caused by the missense mutation in exon 3 that affects several *Spef2* isoforms. SPEF2 has been shown to interact with IFT-related protein IFT20, suggesting the involvement of SPEF2 in IFT^30^. In this study, we generated a mouse model with a stop codon located after exon 2 of *Spef2* gene to further investigate the role of SPEF2 in ciliated tissues in mice.

## Results

### Disruption of *Spef2* gene causes hydrocephalus and growth retardation

For identification of the role of SPEF2 in mice, we have generated a *Spef2* KO mouse model. The targeting construct was designed to produce two mouse lines; conventional full KO model by introducing a DsRed reporter and transcription termination sequence after the *Spef2* exon 2 and a conditional KO model by introducing l*oxP* sites to surround exons 3–5 (Supplemental Fig. S1A). To generate the conditional male germ cell-specific *Spef2* KO mouse line, the *Dsred* construct was removed using FLP-FRT recombination, and subsequently, the mice with floxed *Spef2* gene were crossed with transgenic mice expressing Cre under the *Neurogenin3* (*Ngn3*) promoter^31^. Here we report the results from the conventional KO mouse model, in which the *Spef2* gene was inactivated in all tissues examined. The position of the introduced construct in relation to known *Spef2* transcript variants and other reported *Spef2* mutants is shown in Supplemental Fig. S1B. The genotype of pups was confirmed by PCR using primers flanking the LoxP site after exon 5 to amplify 478 bp product from mutated allele and 379 bp product from wild type (WT) allele (Supplemental Fig. S1A).

In control mice, the 5′ end of the *Spef2* messenger RNA (mRNA) was expressed in the testis, epididymis, brain, trachea, pituitary gland, lung, kidney and eye as demonstrated by RT-PCR using primers amplifying exons 1–3 (Supplemental Fig. S2A). These primers failed to amplify the WT product in any of the *Spef2* KO tissues, therefore confirming the successful abolishment of *Spef2* gene expression (Supplemental Fig. S2A). The expression of the 3′ end of *Spef2* (exons 37–43) was also shown to be absent in KO tissues, while the expression was detected in WT mice in the testis and trachea and at low level in the lung, pituitary gland and kidney (Supplemental Fig. S2A). In the targeted allele, *Dsred* was introduced after *Spef2* exons 1–2 in order to produce a SPEF2(exons1–2)/DsRed fusion protein. However, we were unable to detect the DsRed protein expression in KO tissues (data not shown). Although the exon 3 is, as a rule, not included in the *Spef2(exon1–2)/Dsred* hybrid transcript due to the transcription termination site after the *Dsred* sequence, we were able to detect a low level of the longer *Spef2/Dsred* hybrid transcript in the trachea with exons 1–3 amplifying primers when we used a high number of cycles and a long elongation time in RT-PCR (Supplemental Fig. S2B). The sequencing of this PCR product revealed that the *Dsred* sequence was in fact not in frame with the *Spef2* coding sequence and an additional stop codon was introduced after exon 2 of *Spef2* (Supplemental Fig. S2B). Therefore, the DsRed protein expression could not be used to monitor the tissue distribution of *Spef2* expression.

*Spef2* KO mice suffered from severe hydrocephalus (Supplemental Fig. S3A) and mice survived a maximum of five weeks. The hydrocephalus was present in mice with 129 and B6 background indicating that *Spef2* is a causative gene for PCD^29,32^. The severe hydrocephalus developed between P15 and P30 probably causing the death of majority of mice approximately at three weeks of age. Birth weights of WT and *Spef2* KO mice were comparable, but a difference in weight gain was observed between WT and *Spef2* KO mice during the growth showing a significant difference at P31 (Supplemental Fig. S3B,C). Because SPEF2 has been shown to function in cilia-related processes, we analyzed the structure of cilia in *Spef2* KO mice. The cilia appeared intact, since no differences were observed in cilia length or axonemal structure in *Spef2* KO mice compared to WT (Supplemental Fig. S3D,E).

SPEF2 has been reported to play an essential role during spermatogenesis^28,29^. Because *Spef2* KO mice died before reaching sexual maturity, we were unable to study the progress of spermatogenesis and fertility in *Spef2* KO mice. The analysis of *Spef2* heterozygous (HEZ) mice revealed that the haploinsufficiency of *Spef2* gene did not affect male fertility. *Spef2* HEZ males produced average litter sizes (average number pups/litter 9.5 SD ± 2.13) and the distribution of genotypes of pups followed Mendelian ratio (Supplemental Fig. S3F). We did not find major defects in the overall organization of the seminiferous epithelium and spermatogenesis (Supplemental Fig. S3G). Furthermore, the motility of *Spef2* HEZ spermatozoa was normal and equal numbers of progressive motile (PR), non-progressive motile (NP) and immotile (IM) spermatozoa were counted from both WT and *Spef2* HEZ sperm samples (Supplemental Fig. S3H). Furthermore, the HEZ mice appeared viable and no phenotypic changes were observed compared to WT. Thus, a single copy of the *Spef2* gene appears to be sufficient for the function of the gene.

### *Spef2* is expressed in the bone and cartilage

**Figure 1:**
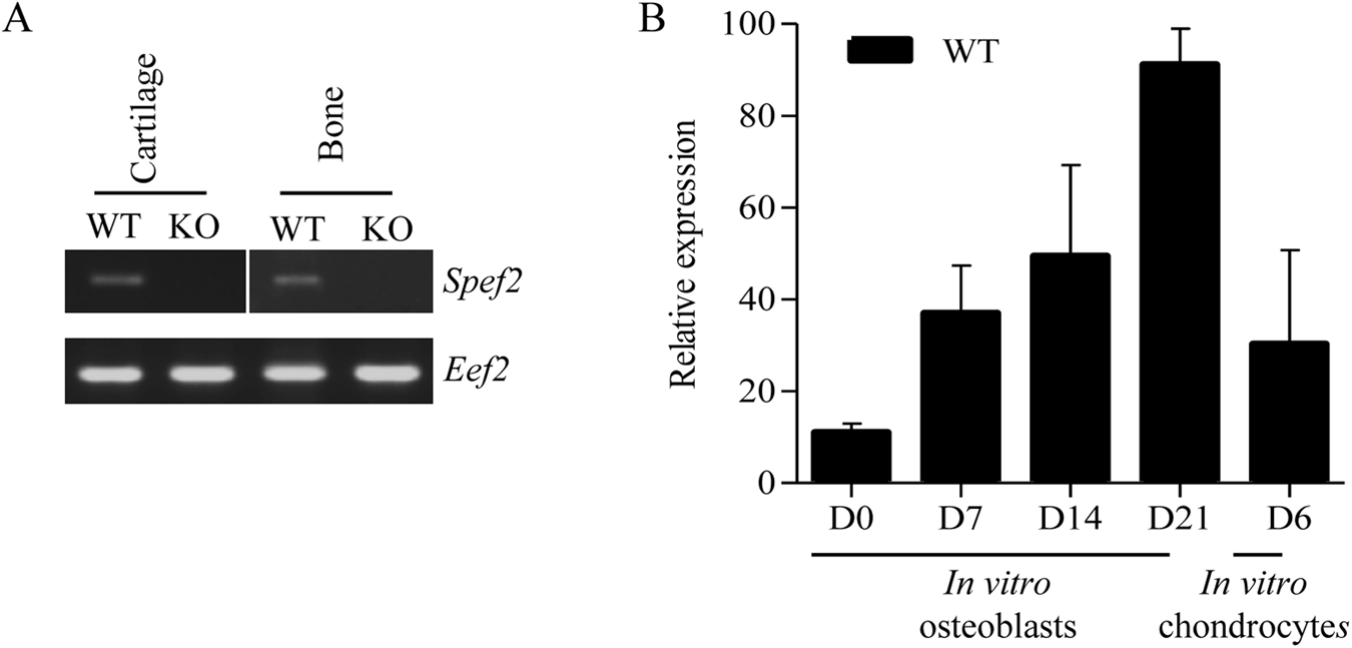
*Spef2* is expressed in the bone and cartilage. (**A**) *Spef2* RNA expression was detected in the bone and cartilage tissues and significantly decreased in *Spef2* KO mice. RNA was extracted from the proximal tibia trabecular bone and cartilage and the same exposure was used for different gels. (**B**) *Spef2* expression was increasing during the osteoblast differentiation *in vitro* and also present in chondrocytes. Primers used for *Spef2* RT-PCR (**A**) and qPCR (**B**) are located in exon 1 and exon 3. Error bars are presented as ± SD.

The size difference between WT and *Spef2* KO mice prompted us to further investigate the possible effects of *Spef2* depletion on bone formation and structure. To study the *Spef2* gene expression in skeletal tissues, we isolated trabecular bone (P31) and cartilage (P3, P15) from proximal tibia. *Spef2* was shown to be expressed in these tissues, and the expression was markedly decreased in both the bone and cartilage of *Spef2* KO mice compared to WT (Fig. 1). To confirm *Spef2* expression in osteoblasts and chondrocytes, we isolated and cultured osteoblasts and chondrocytes from the WT mouse calvaria and ribcages, respectively. *Spef2* gene expression increased during osteoblast differentiation (Fig. 1B) and expression was also detected in the cultured chondrocytes (Fig. 1B).

### Disruption of *Spef2* affects the skeletal growth, structure and strength

**Figure 2:**
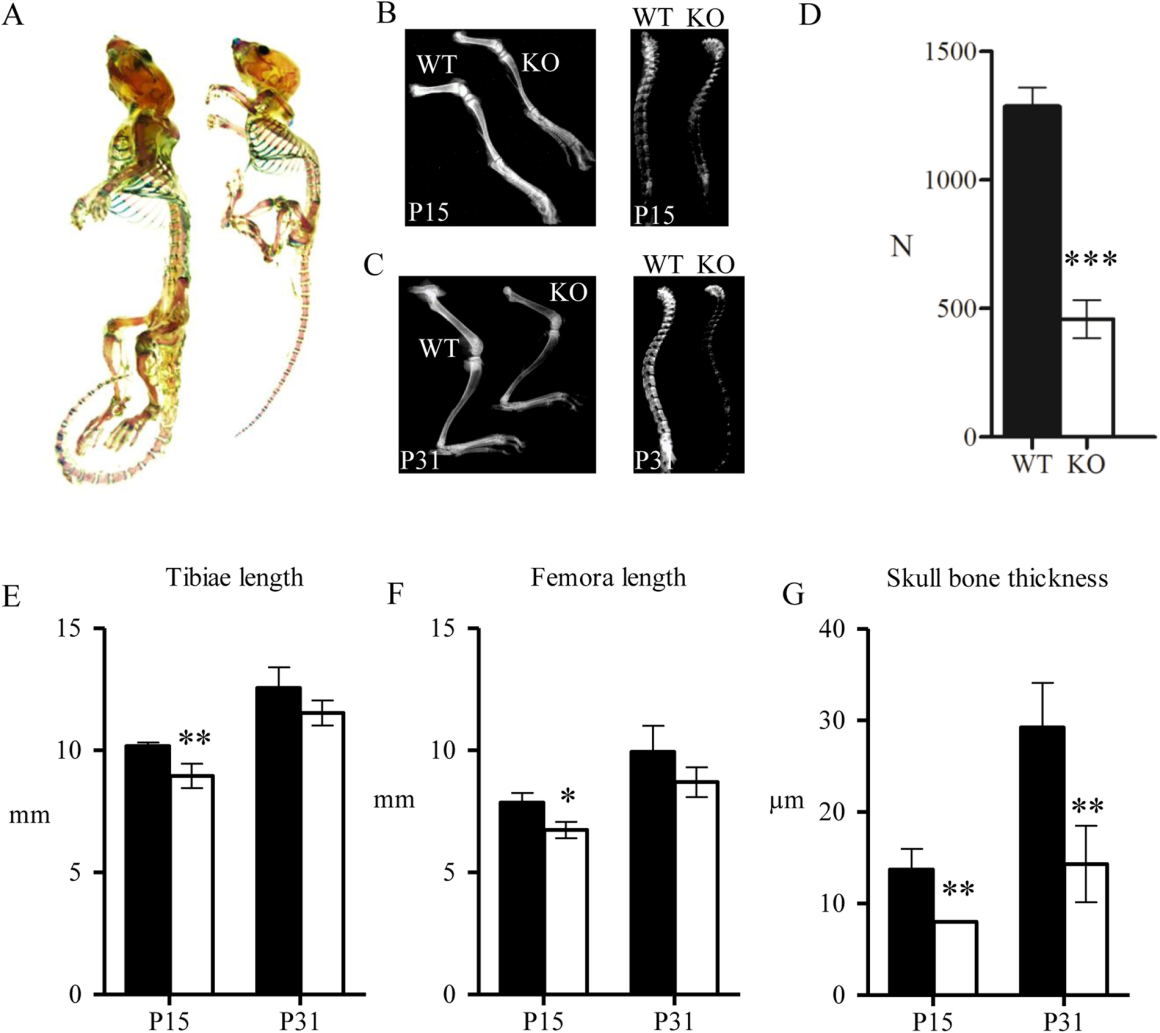
Bone formation is impaired in *Spef2* KO mice. (**A**) Whole-mount staining of the skeleton at P31 presented a drastic size difference, although the proportions of bone and cartilage were similar in WT and *Spef2* KO mice (n = 1). Alizarin red was used to stain the bone and Alcian blue the cartilage tissue. (**B**,**C**) Bone mineral content appears to be decreased in the *Spef2* KO mice compared to WT based on the X-ray images of P15 (**B**) and P31 (**C**) of WT and *Spef2* KO mice hind limbs and spines. (**D**) Left femora mid-shaft was subjected to 3-point bending test and the results show mechanically weaker bone in *Spef2* KO mice. (**E**,**F**) The tibiae (**E**) and femora (**F**) were significantly shorter already at P15 in *Spef2* KO mice. (**G**) The skull thickness was significantly lower at P15 and P31 in *Spef2* KO mice when compared to WT. Error bars ± SD; * = p < 0,05; ** = p < 0,01; *** = p < 0,001.

We analyzed macroscopic organization and composition of the cartilage and bone tissue between WT and *Spef2* KO mice using whole-mount staining. Alcian blue was used to stain the cartilage and Alizarin red the bone tissue. Staining appeared similar in WT and *Spef2* KO mice at P31 and no differences were observed in ossification between WT and *Spef2* KO, although the size difference of the animals was obvious (Fig. 2A). It has to be noted that the staining was performed for only one KO and WT animal. To investigate the bone tissue content in more detail, X-ray images were obtained from hind limbs and spines at P15 and P31 (Fig. 2B,C). X ray images showed a reduced mineral content in *Spef2* KO bone at both vertebral column and in long bones at both timepoints (P15 and P31) compared to WT. Bone strength was further evaluated using a 3-point bending test, where mechanical load was subjected to the WT and *Spef2* KO femora midshaft at P31 (Fig. 2D). *Spef2* KO bones were weaker and broke down with significantly lower maximal load than the WT femurs (Fig. 2D). To investigate whether *Spef2* has a role in long bone growth we measured tibia and femora lengths at P15 and P31 and demonstrated that in *Spef2* KO mice, both bones were significantly shorter at P15 with a (non-significant) growth delay also at P31 (Fig. 2E,F). We also measured the skull bone thickness at P15 and P31 and observed significantly thinner bones at P15 and P31 in *Spef2* KO mice (Fig. 2G). However, since the hydrocephalus was more severe at P31, it may explain the more profound difference between WT and *Spef2* KO at P31 compared to P15.

### Disruption of *Spef2* impairs the cortical and trabecular bone volume and structure

**Figure 3:**
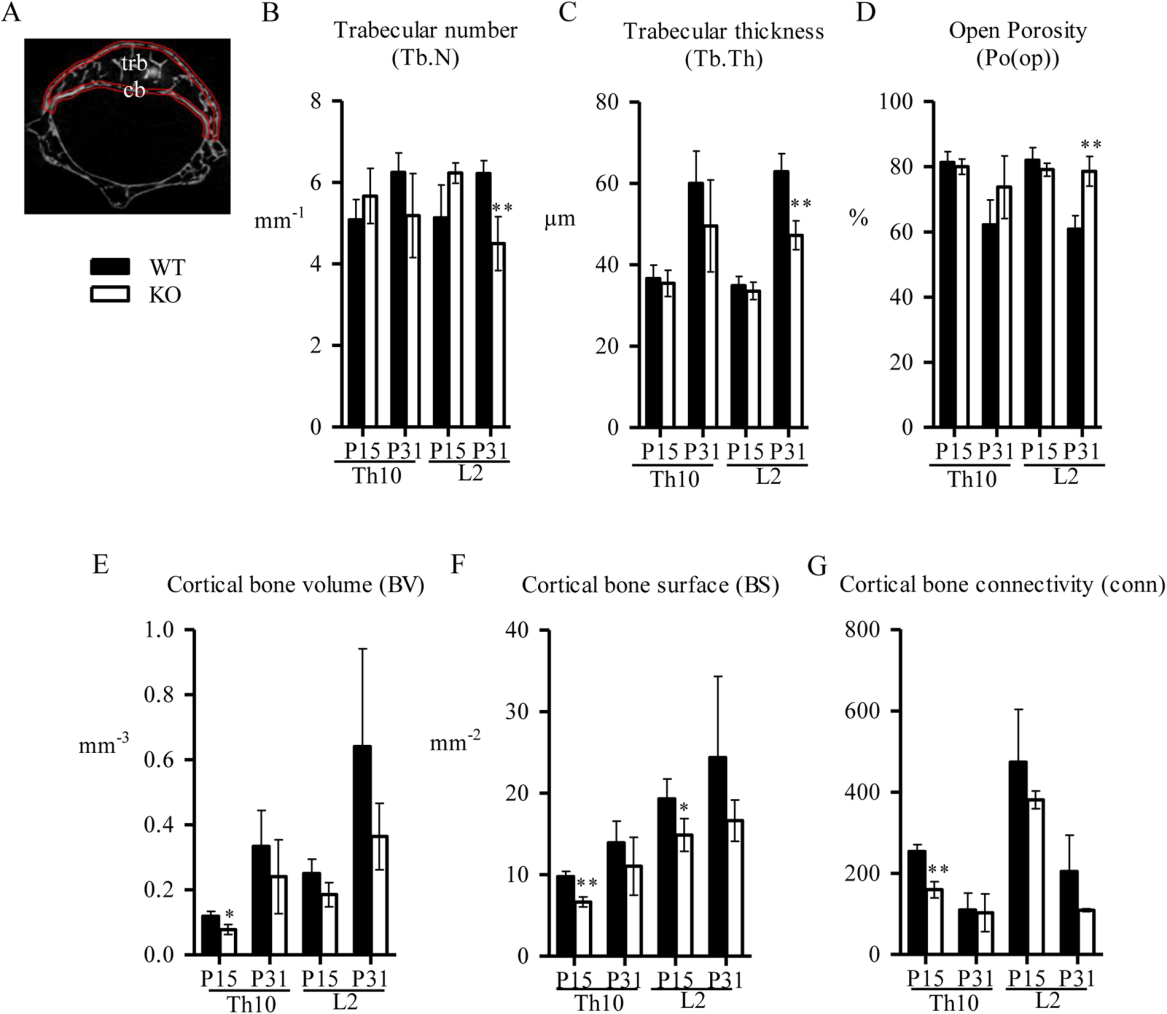
µCT analysis of the trabecular and cortical bone in Th10 and L2 vertebrae. (**A**) Regions of Interest (ROIs) of the trabecular (tbr) and cortical (cb) vertebrae are marked. (**B**–**D**). Trabecular number (Tb.N) (**B**), thickness (Tb.Th) (**C**) and open porosity (Po(op)) (**D**) indicate that depletion of *Spef2* affects the trabecular bone content and structure. (**E**–**G**) Cortical bone volume (BV) (**E**), surface (BS) (**F**) and connectivity (conn) (**G**) suggests decreased bone volume and structure in the *Spef2* KO vertebra. Error bars ± SD; * = p < 0,05; ** = p < 0,01; *** = p < 0,001.

Micro-computed tomography (µCT) was used for microstructural analysis of trabecular and cortical bone in Th10 and L2 vertebrae (Fig. 3A). Trabecular bone volume density (BV/TV) was decreased at P31 in both Th10 and L2 vertebrae (Table 1). Trabecular number (Tb.N, Fig. 3B) and thickness (Tb.Th, Fig. 3C) were significantly decreased and open porosity (Po(op), Fig. 3D) was increased in *Spef2* KO mice vertebrae at P31. Cortical bone volume (BV, Fig. 3E), surface (BS, Fig. 3F) and connectivity (conn, Fig. 3G) appeared reduced in *Spef2* KO mice in both studied vertebrae and timepoints, suggesting more loose cortical bone in the spine.

**Table 1:** µCT data from vertebra Th10 and L2 trabecular bone at P15 and P31.

	WT, P15	KO, P15	WT, P31	KO, P31
**Th10**				
Tissue volume (TV), mm^3^	0,581 ± 0,0237	0,328 ± 0,0466^c^	0,087 ± 0,2623	0,565 ± 0,1641
Bone volume (BV), mm^3^	0,109 ± 0,0235	0,066 ± 0,0098^a^	0,341 ± 0,1493	0,158 ± 0,0872
Bone volume density (BV/TV), %	18,735 ± 3,3512	20,055 ± 2,3983	37,800 ± 7,6047	26,283 ± 9,5989
**L2**				
Tissue volume (TV), mm^3^	1,1436 ± 0,091	0,698 ± 0,1369^b^	1,637 ± 0,6347	0,917 ± 0,113
Bone volume (BV), mm^3^	0,204 ± 0,0317	0,147 ± 0,0389	0,648 ± 0,2709	0,199 ± 0,0591^a^
Bone volume density (BV/TV), %	18,010 ± 3,8619	20,958 ± 1,9395	39,191 ± 4,1460	21,428 ± 4,5726^b^

Error bars ± SD, ^a^ = p < 0,05; ^b^ = p < 0,01; ^c^ = p < 0,001.

**Figure 4:**
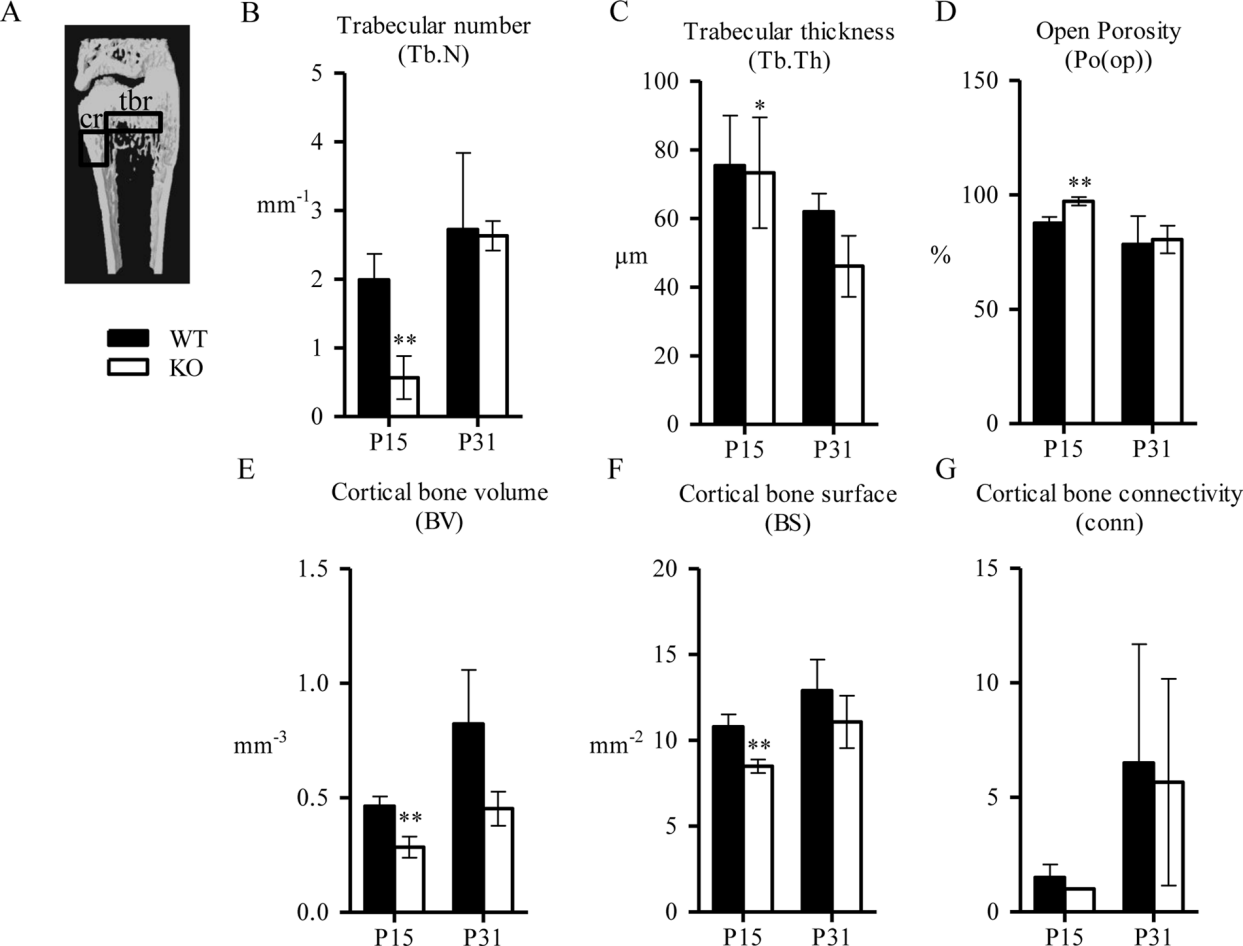
µCT analysis of the distal femur trabecular and cortical bone. (**A**) ROI for the trabecular bone (tbr) was drawn starting from the distal growth plate of the femur, extending for 1 mm towards diaphysis, excluding the cortical bone material. ROI for the cortical bone (cr) was drawn starting from 1 mm from the distal growth plate to the diaphysis, extending a longitudinal distance of 1 mm. (**B**–**D**) Trabecular number (Tb.N) (**B**), thickness (Tb.Th) (**C**) and open porosity (Po(op)) (**D**) indicated reduced trabecular bone content and impaired structure. (**E**–**G**). Cortical bone volume (BV) (**E**), surface (BS) (**F**) and connectivity (conn) (**G**) showed decreased cortical bone volume and structure in *Spef2* KO femurs. Error bars ± SD; * = p < 0,05; ** = p < 0,01; *** = p < 0,001.

Distal femur cortical and trabecular bone parameters were also analyzed using µCT (Fig. 4A). BV/TV was significantly decreased at P15 in distal femur trabecular bone (Table 2). In addition, Tb.N (Fig. 4B) and Tb.Th. (Fig. 4C) were also significantly decreased at P15. Open porosity was increased and difference was significant at P15 (Fig. 4D). Parameters for distal femur cortical bone supported the data acquired from the vertebrae: BV (Fig. 4E), BS (Fig. 4F) and connectivity (Fig. 4G) were all decreased in *Spef2* KO mice.

**Table 2:** µCT data from distal femur trabecular bone at P15 and P31.

	WT, P15	KO, P15	WT, P31	KO, P31
Tissue volume (TV), mm^3^	1,751 ± 0,1775	1,070 ± 0,0802^b^	2,022 ± 0,3248	2,973 ± 1,6425
Bone volume (BV), mm^3^	0,217 ± 0,0507	0,031 ± 0,0179^b^	0,472 ± 0,2936	0,661 ± 0,5184
Bone density volume (BV/TV), %	12,386 ± 2,3503	2,805 ± 1,4952^b^	21,635 ± 10,7071	19,532 ± 4,8941

Error bars ± SD, ^a^ = p < 0,05; ^b^ = p < 0,01; ^c^ = p < 0,001.

### Osteoblast differentiation is impaired in *Spef2* KO mouse

**Figure 5:**
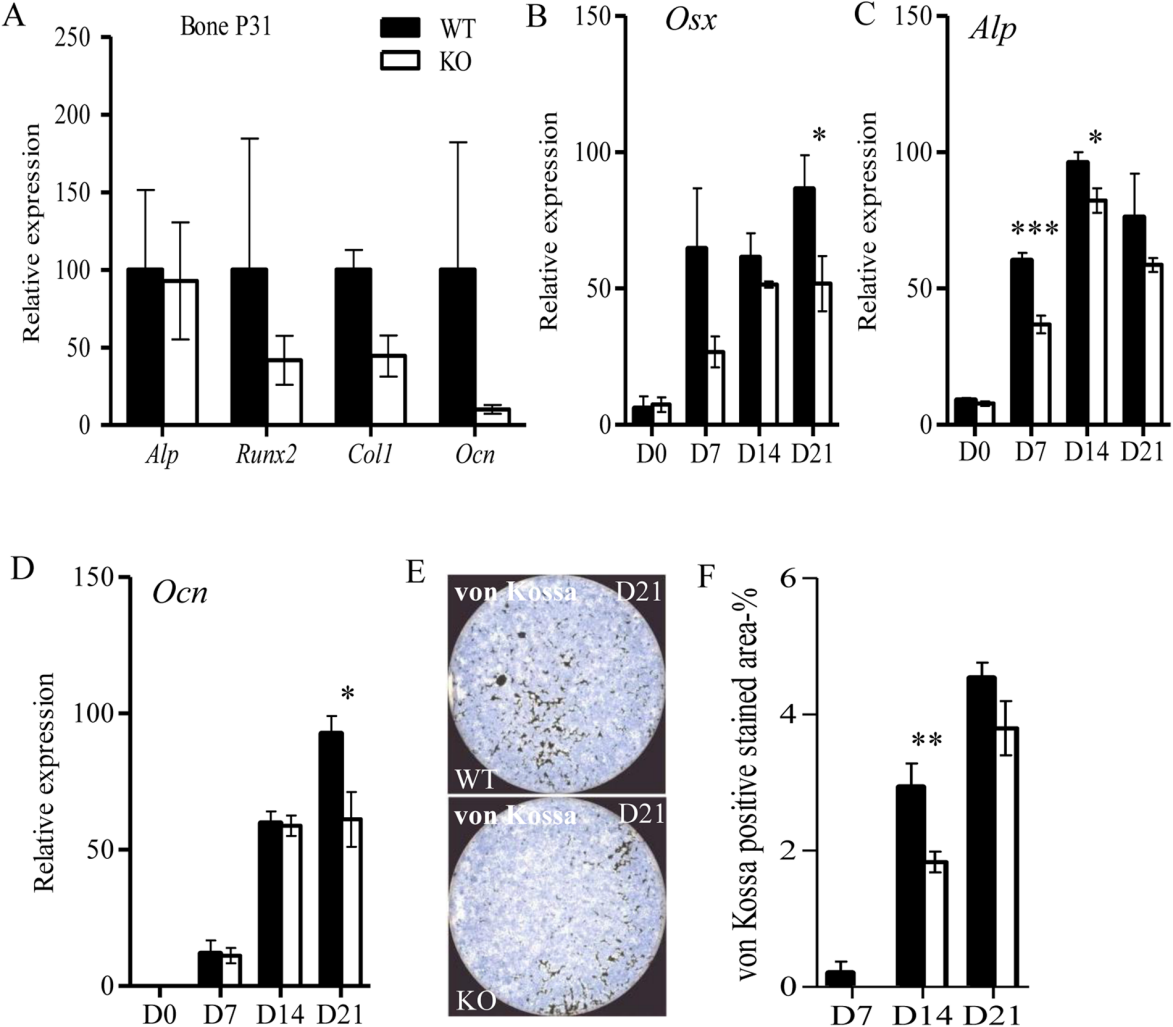
Osteoblast differentiation is impaired in the *Spef2* KO. (**A**) *Alp, Runx2, Col1*, and *Ocn* expressions were measured from RNA isolated from proximal tibia trabecular bone at P31. (**B**) *Osx* as well as (**C**) *Alp* expressions were lower in *Spef2* KO osteoblasts compared to WT osteoblasts during the *in vitro* differentiation. (**D**) Osteoblasts isolated from the *Spef2* KO also presented lower *Ocn* expression and (**E**,**F**) less von Kossa positively stained area indicating lower mineralization capacity. Error bars ± SD; * = p < 0,05; ** = p < 0,01; *** = p < 0,001.

To investigate the effects of *Spef2* depletion on osteoblast differentiation we first analyzed several osteoblast markers of the proximal tibia trabecular bone: the expression of *Alp*, *Ocn*, collagen 1 (*Col1*), *Runx2* and *Osx* was studied by RT-qPCR. Although *Alp* expression appeared unchanged between WT and *Spef2* KO bone tissue, *Runx2, Ocn and Col1* were reduced in *Spef2* KO bone indicating possible defects in osteoblast activity (Fig. 5A). To study the direct role of *Spef2* in osteoblast differentiation, calvarial osteoblasts were isolated from WT and *Spef2* KO mice and cultured for 21 days *in vitro*. Samples were collected at D0, 7, 14 and 21 for *Osx*, *Alp*, and *Ocn* gene expression analysis. Interestingly, the expression of all studied genes was decreased in *Spef2* KO osteoblasts during the *in vitro* differentiation (Fig. 5B–D). Decreased *Osx* expression (Fig. 5B) suggests a defect in early osteoblast differentiation and the decreases in *Alp* (Fig. 5C) and *Ocn* (Fig. 5D) indicate lower mineralization capacity of the *Spef2* KO osteoblasts. These results were supported by the decreased von Kossa staining in *Spef2* KO osteoblast cultures (Fig. 5E,F).

We also analyzed the expression of cathepsin K and tartrate-resistant acid phosphatase in WT and *Spef2* KO bones and did not detect any significant differences suggesting that the differentiation and function of osteoclasts was not affected by *Spef2* depletion (data not shown). This was further supported by similar expression levels of receptor activator of nuclear factor kappa-B ligand and osteoprotegerin in calvarial osteoblasts isolated from WT and *Spef2* KO (data not shown). Altogether these results suggest that the defective bone formation in *Spef2* KO mice is not caused by impaired osteoclast differentiation.

Since bone growth was impaired in *Spef2* KO mice, we analyzed the expression levels of several markers for chondrocyte differentiation. The expression of mineralization marker, *Ocn*, as well as the expression of Collagen 2a1 (*Col2a1*), *Sox9*, *Runx2* and *Col1* was studied in the growth plate cartilage of proximal tibia at P3 and P15, but no significant differences were observed between WT and *Spef2* KO cartilage (Supplemental Fig. S4). However, a slight decrease in *Ocn* expression was detected in *Spef2* KO mice compared to WT at P15. Although we did not observe any major defects in chondrocyte function, it has to be noted that *Spef2* mRNA was shown to be expressed in cultured chondrocytes (Fig. 1B). Thus, more careful analysis is required to conclude about the potential role of *Spef2* in chondrocytes.

## Discussion

Previous studies have indicated the importance of SPEF2 in ciliated tissues, especially during sperm development, tracheal cilia beating and in ependymal cilia function. In this study, we show for the first time that SPEF2 is also required for bone formation. In our mouse model with the full inactivation of *Spef2* gene caused by the introduction of a stop codon after exon 2, we observed severe postnatal growth retardation in addition to previously identified PCD phenotypes. The phenotype of the *Spef2* KO is more pronounced than the phenotype of *bgh* mice that is caused by a missense and nonsense mutation in *Spef2* gene. SPEF2 protein is known to have various splicing variants (Supplemental Fig. S1B), of which all N-terminal splicing variants are eliminated in *Spef2* KO mice. This may explain the phenotypic differences between the different *Spef2* mutant mouse models, and it is likely that the depletion of short N-terminal variants in addition to the full length *Spef2* causes the bone phenotype. The sperm tail phenotype appears to be identical in all *Spef2* mutant animal models^28,29,31^ and is most probably caused by the lack of the full length isoform of SPEF2 protein. This study underlines the importance of the N-terminal part of SPEF2 in various tissues including tissues with primary cilia. Although depletion of SPEF2 appears to have a clear effect only on the structure of the sperm tail axoneme, it obviously does have a role in cilia motility^29^ and potentially in signaling pathways in primary cilia.

The clear changes in various skeletal parameters in *Spef2* KO mice suggest that SPEF2 has a role in bone formation. Although we cannot rule out the possibility that some of the growth defects originate as secondary effects of severe PCD symptoms such as hydrocephalus, several findings suggest that SPEF2 is directly involved in the regulation of bone formation. First, we showed that *Spef2* is expressed in the bone and cartilage, and *Spef2* expression in osteoblasts increases during *in vitro* differentiation. The direct role of SPEF2 is further supported by defective *in vitro* differentiation of *Spef2* KO osteoblasts. The osteoclast function appeared unaffected indicating that the observed phenotype was not due to imbalanced bone remodeling. Furthermore, although *Spef2* was expressed in the cartilage we were unable to observe any significant differences in the expression of specific markers for chondrocyte differentiation. This suggests that the mineralization defect observed in *Spef2* KO mice originates mostly from the defective osteoblast function rather than impaired chondrocyte differentiation and function. However, additional studies are required to confirm the exact role of SPEF2 in cartilage formation.

The effect of SPEF2 depletion in osteoblasts is evident, but the mechanism how SPEF2 regulates osteoblast function and the process of bone formation requires further studies. The existing evidence supports the hypothesis, that the SPEF2 function is associated with cilia-related processes. We have shown that the cilia structure appears intact in *Spef2* mutant models, and therefore, the phenotype is not caused by the absence of cilia (Supplemental Fig. S3)^29^. On the other hand, cilia motility was altered in *Spef2* mutant mice, suggesting that SPEF2 is required for the normal function of cilia^29^. Interestingly, recent data strongly suggests that SPEF2 functions in the intracellular transport of proteins or vesicles via microtubules. SPEF2 interacts with intraflagellar transport protein IFT20 and colocalizes with IFT20 in differentiating male germ cells^30^. Furthermore, we have shown that SPEF2 interacts with a motor protein Dynein 1, and Dynein activity is required for the correct localization of SPEF2 in elongating spermatids^31^. Thus, SPEF2 may have a role in central protein transport or cilia-related signaling pathways required for osteoblast differentiation.

In addition to the canonical role the SPEF2 interaction partner IFT20 in cilia-related transport, it has been shown to function in intracellular trafficking between different cellular compartments^30,33–37^. For example, during craniofacial skeletal development, IFT20 has been shown to be involved in the intracellular trafficking of procollagen from the endoplasmic reticulum (ER) to the Golgi complex^25^. IFT20 depletion caused a severe delay in the exocytosis of the matrix protein collagen 1 from the osteoblasts leading to osteopenia^25^. Moreover, IFT20 has been shown to mediate polycystin-2 (PKD2) trafficking to cilia from the ER through the Golgi complex^38^. Osteoblast specific depletion of *Pkd2* has been shown to results in similar defects in bone mineral density, trabecular bone volume and cortical thickness, and expression of osteoblast related genes e.g. *Ocn* and *osteopontin*^38^ that were observed in *Spef2* KO mice. On the basis of these studies, it is possible that SPEF2 and other cilia- and IFT-related genes have diverse roles in osteoblasts, both in cilia and non-cilia associated sites. Even though the detailed molecular mechanisms of SPEF2 function in osteoblasts remain to be characterized, our results have revealed a novel important role of SPEF2 in the bone formation and mineralization in mice.

## Material and Methods

### Generation of *Spef2* full knockout mouse model

Mice BAC clone (RP23-340E4) containing exons 3-5 (including 3′ and 5′ -flanking regions) from *Spef2* gene (chromosome 15) was purchased from Children’s Hospital Oakland Research Institute (Oakland, CA, USA). All primers used for targeting construct are listed in Supplemental Table S1. Shaving part containing ampicillin resistance and 50 bp homology arms were cloned from pACYC177 plasmid. Shaving part was electroporated into electrocompetent *E.Coli* cells containing BAC clone and pRedET for recombination. LoxP-PGK-tn5-neo-loxP –cassette was cloned from pGKneo10xp plasmid and recombined into Shaved BAC clone after exon 5. Neo cassette was removed from the shaved BAC clone using 294-Cre *E.coli* cells leaving only one LoxP site after exon 5. LoxP, Frt and DsRed2 were cloned from pIRES2-DsRed2 plasmid. Targeting vector was digested with NheI, SpeI, ScaI and BglII (Promega Corporation, Madison, WI, USA) according to manufacturer’s instructions to confirm the correct orientation and insertion of all cloned inserts. *Spef2* targeting vector was linearized using Sac II restriction enzyme (Promega, Madison, WI, USA). Linearized *Spef2* targeting vector was electroporated into hybrid mouse embryonic stem cells (G4, 129S6B6F1) and homologous recombination was screened by PCR. To generate chimeric mice, ES cells were injected into blastocysts of C57BL/N6 mice and targeting vector integration to the genome was detected from the DNA samples of pups isolated from ear marks. Primer sequences for *Spef2* genotyping PCR are listed in Supplemental Table S1. Three chimeric male mice were born and were bred with C57BL/6NHsd females. Two founders transferred the transgene into the next generation. Mice used for further analysis originated from heterozygous breedings (N1) and were maintained as inbred for several generations. Control mice for the experiments were used WT mice originating from *Spef2* heterozygous breedings. WT and *Spef2* HEZ mice presented normal health status while *Spef2* KO mice presented the phenotype caused by depletion of the *Spef2* gene.

### Ethical statement

Mice were sacrificed with CO_2_ or cervical dislocation and thereafter tissues were collected for all experiments. All mice were maintained in a specific pathogen-free stage at the Central Animal Laboratory of the University of Turku and handled in accordance with international guidelines on the care and use of laboratory animals. Studies were approved by the Finnish ethical committee for experimental animals (license 315/041003/2011).

### Animal material

For all experiments male mice were used except for *in vitro* studies, where collected material was pooled from animals with a specific genotype. Age matched WT mice were used as controls from the same litter, when possible. All experiments were repeated (number of animals used is indicated for each experiment) except the whole-mount preparation that was done only for one animal per genotype.

### Sperm motility analysis

The cauda epididymis of WT (n = 8) and *Spef2* HEZ (n = 10) mice (8–9 week old) was dissected and placed in +37 °C KSOM medium with amino acids (Merck Millipore). Sperm was collected by making small incision to cauda and let them swim out at +37 °C for 30 min. Sperm motility was investigated under microscope, where 100 spermatozoa were counted in every sample and classified as progressive motility (PR), non-progressive motility (NP) or immotile (IM). The average of duplicates was used for analysis.

### Histology of the testis

Testis of adult WT and *Spef2* HEZ mice was dissected and fixed with Bouin’s solution, washed several times with 70% ethanol and embedded in paraffin. Paraffin embedded testis were cut into sections, deparaffinized, rehydrated and stained with Mayer’s Hematoxylin (Histolab, Västra Frölunda, Sweden) and eosin. Sections were mounted using Pertex (Gibco, Thermo Fischer Scientific, Waltham, MA, USA) mounting media.

### Whole-mount preparations

For whole-mount preparations skeletons of P31 male mice (n = 1 for WT and *Spef2* KO) were dissected as whole and fixed in 95% ethanol for four days. The skeletons were stained overnight with Alcian Blue 8 G, washed with ethanol, cleared in 1% KOH for 8 h, stained with Alizarin red for 8 h, and cleared in 2% KOH until the staining was easily observable. The preparations were brought to glycerol in ascending concentrations and photographed on a projection table.

### X-ray analysis and micro-computed tomography

X-ray images of formalin fixed hind limbs (n = 4 for WT, n = 3 for *Spef2* KO) and spinal columns (n = 4 for WT, n = 3 for *Spef2* KO) were obtained by using Faxitron LX-60X-ray imaging device (X-Ray LLC, Lincolnshire, IL, USA). To determine the three-dimensional structure of the bones, femora, thoracic vertebral bone Th10 and lumbar bone L2 vertebrae were imaged by micro-computed tomography (µCT, Skyscan 1072 Micro-CT device, Kontich, Belgium) and the data was reconstructed by using the Nrecon 1.6.9.4 software and modeled and analyzed by CTan 1.13.5.1 software (SkyScan). In µCT analysis image pixel size of 4.18467 μm (P15 group) or 7.32622 μm (P31 group) for vertebral bones, and 9.76926 μm for femora was applied; X-ray tube potential of 70 kV and current of 148 μA were used with integration time of 3900 ms and rotational step of 0.45 degrees. During reconstruction smoothing level of 3, beam-hardening reduction of 85%; and ring artifact reduction level of 7 were used with attenuation coefficient value range of 0.006 to 0.15.

### Mechanical testing

Bone mechanical properties were evaluated by applying 3-point bending test by using Nexygen program (Lloyd Instruments, West Sussex, United Kingdom). Left femora of P31 male mice (n = 4 for WT, n = 3 for *Spef2* KO) were placed on two holders located at a range of 3 mm. The bending force was applied at a crosshead speed of 5 mm/min to the middle until fracture occurred. Maximum load (N) was obtained directly from the load-deformation curve.

### RNA analysis

For mRNA analysis tissues were collected, snap frozen in liquid nitrogen and stored at −80 °C. For gene expression analysis of the proximal tibiae cartilage and trabecular bone mass (n = 2–4 for WT, n = 2–3 for *Spef2* KO) were collected in RNAlater stabilization solution (RNAlater RNA stabilization reagent, Qiagen, Hilden, Germany) and stored at −80 °C. Total RNA was extracted by applying the RNeasy Midi kit (Qiagen) following manufacturer’s instructions.

### Real-time PCR (RT-PCR)

For analysis of gene expression with RT-PCR the total RNA was reverse transcribed with random primers and an RT-PCR kit (ImProm-II Reverse Transcription System, Promega) according to the manufacturer’s instructions. Produced cDNA was amplified by using gene specific primers listed in Supplemental Table S1. Housekeeping gene *Rpl13*or *Eef2* was used as a reference gene to calculate the relative expression. The qPCR was performed with a ViiA™ 7 Real-Time PCR System in 96-well microtiter plates using Absolute qPCR SYBR Green ROX Mix (VWR, Radnor, PA, USA). Amplification by qPCR contained 12.5 μl of Absolute qPCR SYBR Green Mix, 100 ng of cDNA, and 70 nM of each primer in a final volume of 25 μl. Amplifications were initiated with 15 min enzyme activation at 95 °C followed by 40 cycles of denaturation at 95 °C for 15 s, primer annealing at 60 °C for 1 min, and extension at 72 °C for 30 s. All samples were amplified in triplicate, and the mean value was used for further calculations. Raw data were analyzed with the sequence detection software (Applied Biosystems, Foster City, CA, USA) and relative quantitation was performed with GenEx software (MultiD, Göteborg, Sweden). Ratios between the target and reference gene were calculated by using the mean of these measurements. A standard curve for each primer pair was produced by serially diluting a control cDNA and used to correct the differences in amplification. A melting curve analysis was performed allowing single product-specific melting temperatures to be determined. No primer–dimer formations were generated during the application of 40 real-time PCR amplification cycles.

### Sanger sequencing

For Sanger sequencing of the *Spef2* KO hybrid transcript the cDNA fragment was amplified with *Spef2* specific primers in exons 1 and 3 (Supplemental Table S1) and PCR amplicons were purified using ExoSAP-IT (Amersham Biosciences). PCR fragments were sequenced in both directions with amplification primers and Dsred primers within the PCR amplicon (Supplemental Table S1). Sequencing was performed on a MegaBace 500 capillary DNA sequencer (Amersham Biosciences) using DYEnamic ET Terminator Kits with Thermo Sequenase II DNA Polymerase (Amersham Biosciences). The data was analyzed using the Variant Reporter v1.0 program (Applied Biosystems) and Sequencer 5.2.3 (Gene Codes Corporation).

### Calvarial osteoblast culture

Calvarias were collected from three-day old WT and *Spef2* KO mice. After decapitation the calvarias were removed and cells were released from the matrix using 1 ml digestion medium [0.1% Collagenase A (Roche Diagnostics, Germany), 0.2% Dispase II (Roche Diagnostics, Germany) diluted in α-MEM] at 37 °C with shaking. After 10 min digestion first fraction was collected and discarded and four subsequent fractions were collected in every 20 min and pooled. Cells were plated in α-MEM containing 10% FBS and antibiotics (proliferation medium) in 10 cm dishes. Cells were allowed to proliferate until 80–90% confluency before pooling the same genotypes and seeding into six-well plates cultured until confluency (day 0 timepoint). Osteoblast differentiation was induced with 5 mM sodium beta-glycerophosphate, 10^−8^ M dexamethasone and 50 µg/ml ascorbic acid in proliferation medium. Differentiation medium was changed every 2–3 days. Samples for RNA extraction were collected at 0, 7, 14 and 21 days by rinsing the wells with PBS, scraping cells off the bottom of the well with a cell scraper and stored in −20 °C in RNAlater (Invitrogen, USA).

### Cytological stainings and quantification of stained areas in calvarial osteoblast cultures

For cytological stainings, osteoblast cultures were rinsed with PBS and fixed with 3.7% formalin for 10 min. After dH_2_O washes formalin-fixed wells were stained for ALP with Naphthol AS MX-PO_4_ (Sigma, USA) dissolved in DMF (Sigma USA) mixed with Fast Blue RR salt (Sigma, USA) in 0.1 M Tris-HCl (pH 8.3). ALP stained wells were stained for von Kossa with 2.5% Silver nitrate (Fisher Scientific, UK) for 30 min exposed to direct light, and washed with dH_2_O for three times. ALP and von Kossa stained six-well plates were scanned using a flatbed scanner with a transparency adaptor (HP ScanJet 5370 C) and saved as 24-bit color images in TIFF format. Transparency exposure adjustments were maintained constant to create images of equal intensity. Positively stained areas were quantified using Imaging Software ImageJ. RGB images were split into three 8-bit grayscale images containing the red, green and blue components. Threshold and Region of Interest (ROI) were adjusted and kept the same to maintain standard measuring conditions.

### Chondrocyte cultures

Primary chondrocytes were isolated from three-day old mouse using standard protocols^39^. Briefly, chondrocytes were digested from mice ribcages during 2 h digestion in 3 mg/ml collagenase D diluted in Dulbecco’s Modified Eagle Medium (D-MEM) at 37 °C. Digestion solution was changed to 0.5 mg/ml collagenase D in D-MEM and ribcages were incubated at 37 °C for overnight. After filtering through 100 µm strainer, chondrocytes were seeded (3 × 10^6^ cells/well) on six-well plates and cultured in D-MEM supplemented with 100 U/ml penicillin-streptomycin, 10% FBS (Gibco, USA), 2 mM L-glutamine and Insulin-Transferrin-Selenium (ITS, Gibco). The culture medium was changed every 2 days and cells were harvested in RNALater at day 6 for RNA isolation.

### Electron microscopy

Trachea were isolated from WT and *Spef2* KO mice and fixed with 5% glutaraldehyde. Samples were treated with potassium ferrocyanide-osmium fixative and embedded in epoxy resin. Sectioned samples were stained using 1% uranyl acetate and 0, 3% lead citrate. Samples were visualized with JEM-1400 Plus (JEOL).

### Immunohistochemistry of the bone and cartilage

Tibia of WT and *Spef2* KO (n = 3) mice were collected and fixed in 10% buffered formalin overnight, decalcified in 5% formic acid, embedded in paraffin and cut into 5 μm-thick sections. Tibia sections were digested using ficin (Digest-All 1, Thermo Fischer Scientific) at +37 °C for 10 min after paraffin removal and rehydration. Sections were blocked with 10% normal goat serum and 3% bovine serum albumin diluted in 0,01% Triton-X 100 PBS. Primary antibody (anti-acetylated α-tubulin (Sigma-Aldrich, St Louis, MO, USA) 1:1000) was diluted in 3% normal goat serum, 1% bovine serum albumin and 0, 01% Triton-X 100 in PBS and incubated at +4 °C overnight. After washes with 0, 1% Triton-X 100 in PBS sections were incubated with secondary antibody (1:500, goat anti-mouse Alexa 488 (Molecular probes, Eugene, OR, USA)) at room temperature for 1 hour. Sections were mounted with Prolong® Diamond Antifade Mountant (Molecular Probes) and imaged using Leica DMRBE microscope and DFC320 camera.

### Statistical analysis of data

Data is presented as average values with standard deviation (SD). Statistical differences were calculated by paired Student’s t-test was used for pairwise comparisons between groups. P-values of 0.05 or less were considered statistically significant.

## Electronic supplementary material

Supplementary data
