# Supplementary material for: Cilia-related protein SPEF2 regulates osteoblast differentiation
Source: Sci Rep. 2018 Jan 16;8:859. doi: 10.1038/s41598-018-19204-5 (PMC5770417; doi:10.1038/s41598-018-19204-5)
Supplement: Supplementary file 1 — Supplementary data [file 41598_2018_19204_MOESM1_ESM.pdf]

Supplemental information

Cilia-related protein SPEF2 regulates osteoblast differentiation

Mari S. Lehti[1,2], Henna Henriksson[2], Petri Rummukainen[2], Fan Wang[2], Liina Uusitalo-Kylmälä[2], Riku Kiviranta[2,3], Terhi J. Heino[2], Noora Kotaja[2] and Anu Sironen[1,*]

[1]Natural Resources Institute Finland (Luke), Green Technology, FI-31600 Jokioinen, Finland; [2]Institute of Biomedicine, University of Turku, FI-20520 Turku, Finland; [3]Department of Endocrinology, Division of Medicine, University of Turku and Turku University Hospital, FI-20520 Turku, Finland

Supplemental Figure S1

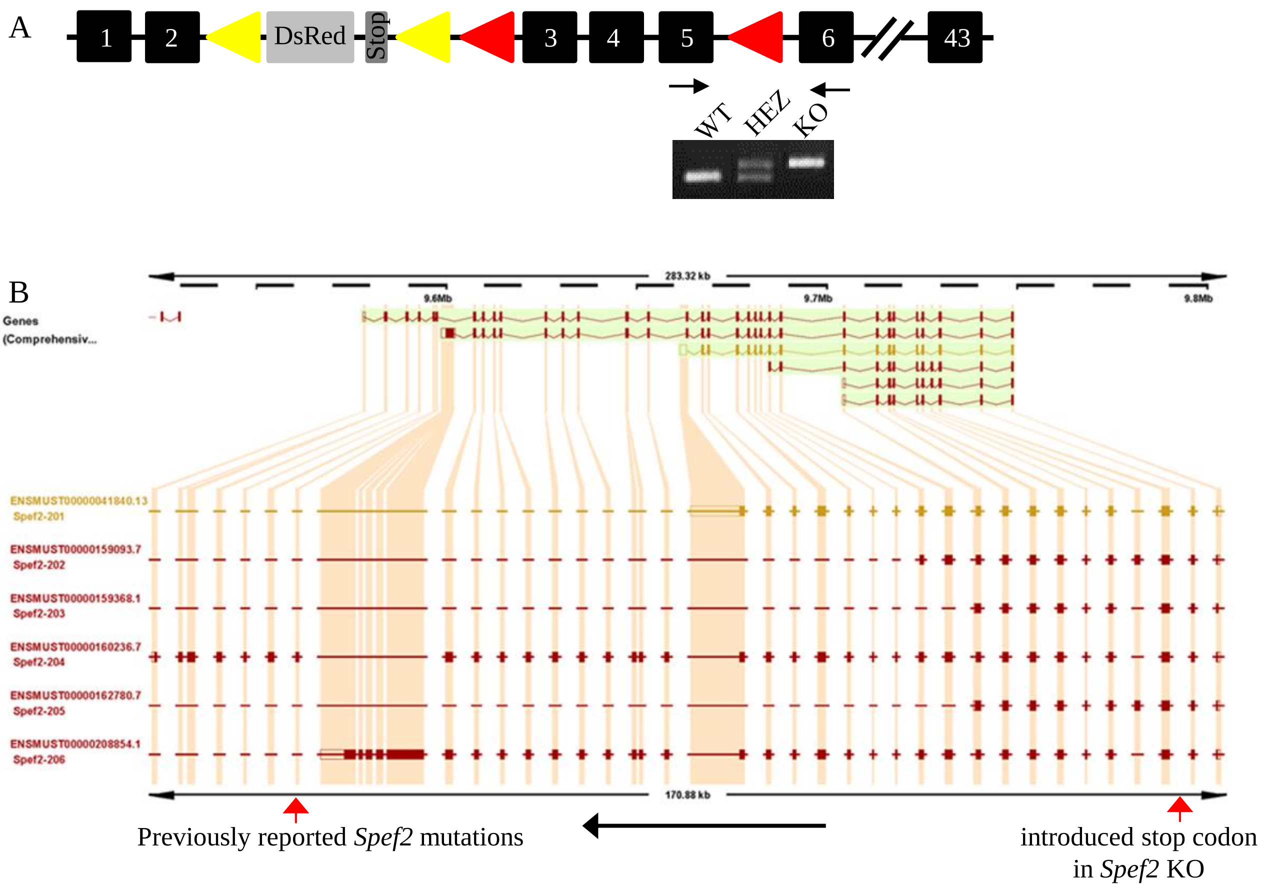

Supplemental Figure S1. **Generation of the *Spef2* KO mouse model.** (A) *Spef2* targeting construct was designed to produce two mouse lines: conventional full KO mouse model by introducing a stop codon after *Spef2* exon 2 and a conditional KO mouse model by introducing of l*oxP* sites (red arrow head) to surround exon 3-5. *Frt* sites (yellow arrow head) surround *Dsred* and the stop codon and were used to remove this part of the construct for creation of the conditional KO mouse model. Genotyping primers were designed to amplify the last *Loxp* site (black arrows). Genotyping PCR produced 379 bp product from the WT allele and 478 bp product from the KO allele. (B) Six protein coding isoforms are predicted for SPEF2 (Ensembl database). Red arrows mark the positions of previously published *Spef2* mutations and the gene trap introduced in this study and the black arrow the direction of transcription.

Supplemental Figure S2

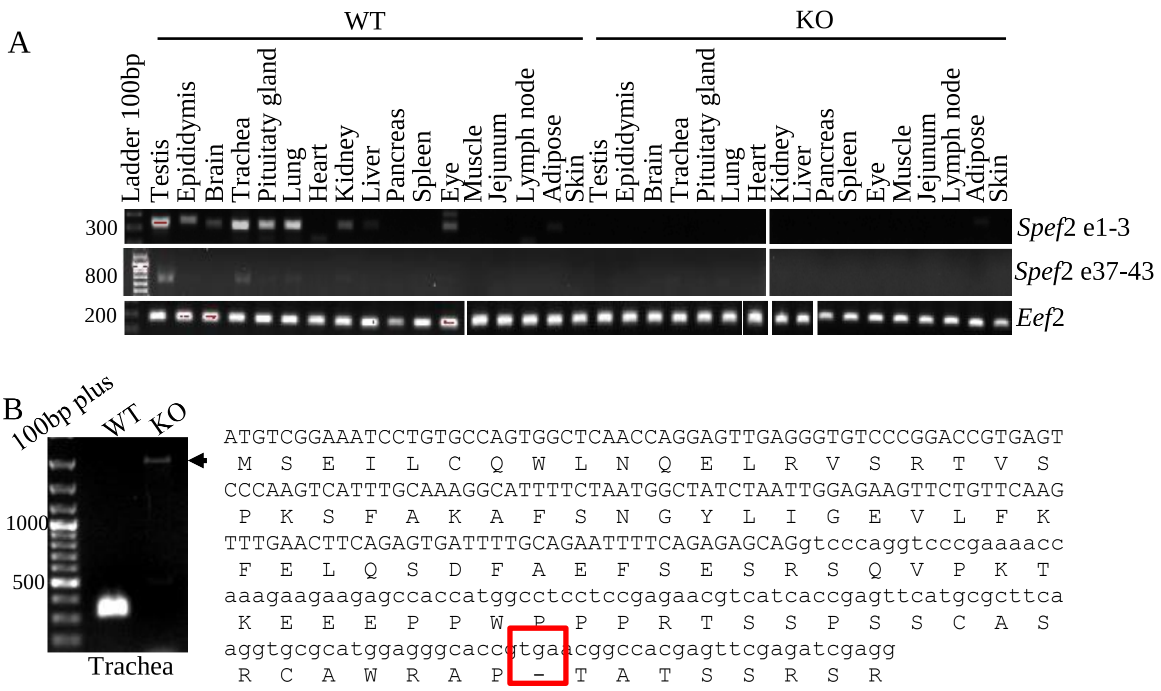

Supplemental Figure S2. **Introduction of the targeting construct in *Spef2* intron 2 resulted in the lack of *Spef2* expression in KO mice**. (A) Expression of *Spef2* exons 1-3 and exons 37-43 was analyzed by RT-PCR in several mouse tissues. The absence of the *Spef2* product in KO tissues confirmed the successful depletion of the *Spef2* gene. *Eef2* was used as an internal control. (B) RT-PCR with the primers amplifying the region from exon 1 to exon 3 revealed the expression of the hybrid *Spef2*/*Dsred* mRNA in the trachea of KO mice (black arrow). Sanger sequencing of this hybrid transcript showed that a stop codon was accidentally introduced in the *Dsred* sequence during the cloning protocol (red box), therefore blocking the expression of DsRed reporter. *Spef2* exon 1-2 sequence is indicated with capital letters, *Dsred* sequence with lower case letters.

Supplemental Figure S3

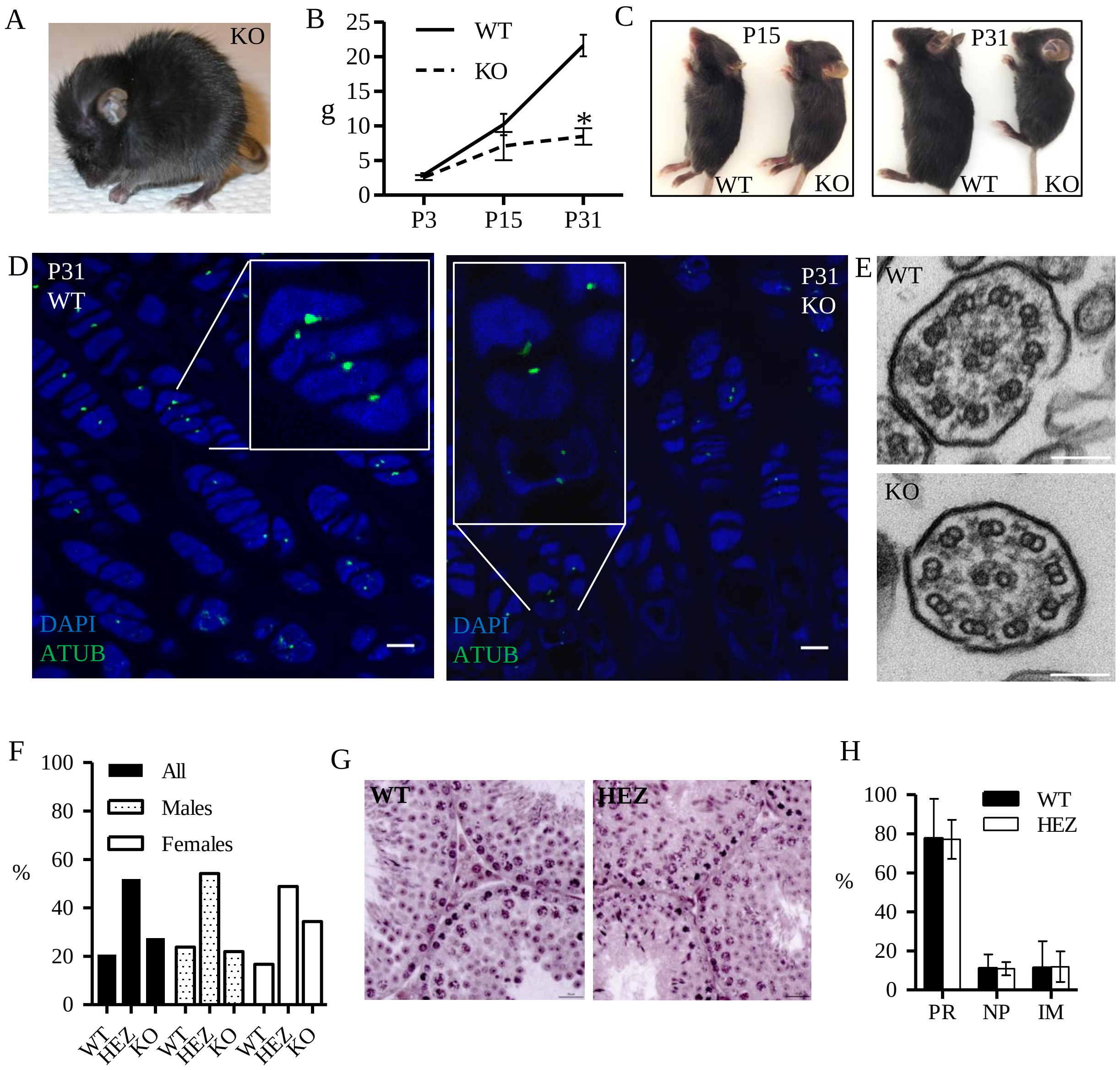

Supplemental Figure S3. Depletion of the *Spef2* gene causes hydrocephalus and growth retardation. (A) *Spef2* KO mice suffer from severe hydrocephalus. (B) The body weight of *Spef2* KO mice was significantly decreased at P31 compared to WT. (C) Growth retardation was observed already at P15 and it was significant at P31. (D) Cilia were present in the tibial growth plate of WT and *Spef2* KO as shown by acetylated alpha-tubulin (ATUB) staining. Scale bar 10 µm. (E) The axonemal structure of tracheal cilia was not affected in *Spef2* KO. Scale bar 100 nm. (F) *Spef2* HEZ breedings produced all genotypes in Mendelian ratio. (G) Organization of the seminiferous tubule was intact in *Spef2* HEZ mice. (H) Sperm motility was not affected in *Spef2* HEZ mice. PR = progressive motility, NP = non-progressive motility, IM = immotile. Error bars ±SD; scale bar = 20 µm; * = $p < 0{,}05$.

Supplemental Figure S4

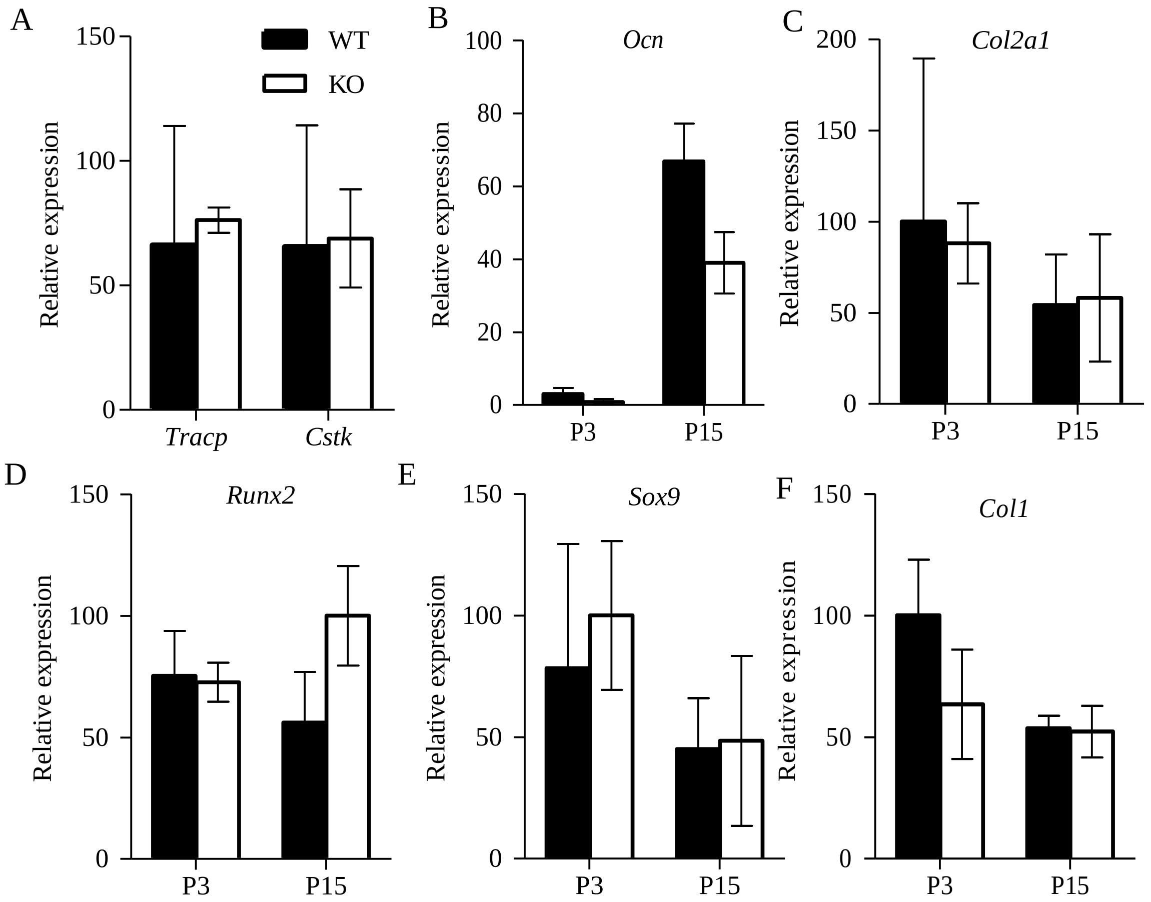

Supplemental Figure S4. *Tracp* and *Cstk* expression in the bone and *Ocn, Col2a1, Runx2, Sox9* and *Col1* expression in the cartilage. (A) *Tracp* and *Cstk* expression in the bone of *Spef2* KO mice was not significantly changed compared to WT at P31. (B) *Ocn* expression was lower in *Spef2* KO mice at P15, when the expression was first clearly detectable. (C-F) Expression in the *Col2a1*, *Runx2*, *Sox9* and *Col1* in WT and *Spef2* KO mice. No significant changes were observed between WT and *Spef2* KO in any analyzed genes. Error bars ±SD.

Supplemental Table S1.

Primers used in the generation of the *Spef2* full knockout mouse model

Shaving	Sense	ATTCTGCTGATCCACGAGTATGGGAGTTCCTTTGTGTGGGCTATTTCATTTGAAGACGAAAGGGCCTC
	Antisense	AACAGAAGTATTGGAAATCTGGTTCCATGCACGTAACCACAGAGTAGTCTGGCAGACCTCAGCGCTAG
Neo-cassette	Sense	AATGTGGCTTCTTATAAATCCCAGAAATGTTAATTGGCTTGTTGCTGTTAGAAGCAGGGATTCTGCAAAC
	Antisense	TGGTCTAGTAAACTATATCTCCAGTGCATGTATTTTAATTTAAAATGTTTGGCGGATTTGTCCTACTCAGG
Dsred	Sense	AGAATGATTCCCATAGCTAACATGATTCAGTTAAATAATGTTGAGAGTTTgaagttcctattctctagaaagtataggaacttc**CCAGCTAACCAGTAACCTCTGCCCTTTCTCCTCCATGACAACCAG**gtcccgaaaaccaaagaagaagaGCCACC*atggcctcctccgagaacgt*
	Antisense	CTAATATCTTAAGTTAATCCTAAATTTTAACTTGACAATAGTTCTTTCATATAACTTCGTATAATGTATGCTATACGAAGTTATAAGCTTGAAGTTCCTATACTTTCTAGAGAATAGGAACTTCGGATTTTGGTCATGA*ATAACTTCGTATAATGTATGCTATACGAAGTTAT*AAGCTTgaagttcctatactttctagagaataggaacttc*ggattttggtcatgagattat*

In primer sequences, homology arms are in capital letters and underlined, the loxP site is in capital letters in italics, the Frt site is in lowercase letters, DsRed is in lowercase letters in italics, the En2_exon2 is in lowercase letters and underlined and the En2_intron1 is in capital letters in bold.

Primers for genotyping

Sense	TAGCAGGATGTAGCTTGAGCCC
Antisense	TAACAGCCCATATCTTGAGTGATG

Primers for Sanger sequencing

Spef2	Sense	CTGGAAAGTTCCTAGCACCTGCAAG
	Antisense	GTTTGGTTGCTGCCCCAGGCTT
Dsred	Sense	CATCCCCGACTACAAGAAGC
	Antisense	TGGTCTTCTTCTGCATCACG

Primers for RT-qPCR

Ribosomal protein L 13a (*Rpl13a*)	Sense	AGGGGCAGGTTCTGGTATTG
	Antisense	CCGAACAACCTTGAGAGCAG
Eukaryotic translation elongation factor 2 (*Eef2*)	Sense	GCTTCCCTGTTCACCTCTGACTCTG
	Antisense	CCTTGCACACAAGGGAGTCGGT
Sperm flagellar protein 2 (*Spef2*) e1-3	Sense	CTGGAAAGTTCCTAGCACCTGCAAG
	Antisense	GTTTGGTTGCTGCCCCAGGCTT
Sperm flagellar protein 2 (*Spef2*) e37-43	Sense	AGGCACCATCACCTACGAAC
	Antisense	GTGCATGCTCACTCCTTTGA
Osterix (*Osx*)	Sense	TCCCATTCTCCCTCCCTCT
	Antisense	GGACTGGAGCCATAGTGAGC
Osteoprotegerin (*Opg*)	Sense	ACCCAGAAACTGGTCATCAGC
	Antisense	CTGCAATACACACACTCATCACT
Receptor activator of nuclear factor kappa-B ligand (*Rankl*)	Sense	TGAAGACACACTACCTGACTCCTG
	Antisense	CCACAATGTGTTGCAGTTCC
Tartrate-resistant acid phosphatase (*Tracp*)	Sense	CGTCTCTGCACAGATTGCAT
	Antisense	AAGCGCAAACGGTAGTAAGG
Cathepsin K (*Cstk*)	Sense	CGAAAAGAGCCTAGCGAACA
	Antisense	TGGGTAGCAGCAGAAACTTG
Osteocalcin (*Ocn*)	Sense	CCCAGACCTAGCAGACACCA
	Antisense	GGGACTGAGGCTCCAAGGTAG
Alkaline phosphatase (*Alp*)	Sense	TCCTGACCAAAAACCTCAAAGG
	Antisense	TGCTTCATGCAGAGCCTGC
Collagen 1 (*Col1*)	Sense	CATGTTCAGCTTTGTGGACCT
	Antisense	TAGGCCATTGTGTATGCAGC
Collagen 2a1 (*Col2a1*)	Sense	GTGTGAAGGGTGAGAGTGG
	Antisense	AGAACCTTGAGCACCTTCAG
SRY-box-9 (*Sox9*)	Sense	TCAGCAAGACTCTGGGCAAG
	Antisense	ACGTCGGTTTTGGGAGTGG
Runt-related transcription factor 2 (*Runx2*)	Sense	CCGCACGACAACCGCACCAT
	Antisense	CGCTCCGGCCCACAAATCTC
